# Adhesion and Self-Healing between Monolayer Molybdenum Disulfide and Silicon Oxide

**DOI:** 10.1038/s41598-017-14921-9

**Published:** 2017-11-07

**Authors:** Seung Ryul Na, Youngchan Kim, Changgu Lee, Kenneth M. Liechti, Ji Won Suk

**Affiliations:** 1 Department of Aerospace Engineering and Engineering Mechanics, The University of Texas at Austin, Austin, Texas 78712 USA; 2 SKKU Advanced Institute of Nanotechnology, Sungkyunkwan University, Suwon, Gyeonggi-do 16419 Republic of Korea; 3 School of Mechanical Engineering, Sungkyunkwan University, Suwon, Gyeonggi-do 16419 Republic of Korea

## Abstract

The adhesion interactions of two-dimensional (2D) materials are of importance in developing flexible electronic devices due to relatively large surface forces. Here, we investigated the adhesion properties of large-area monolayer MoS_2_ grown on silicon oxide by using chemical vapor deposition. Fracture mechanics concepts using double cantilever beam configuration were used to characterize the adhesion interaction between MoS_2_ and silicon oxide. While the interface between MoS_2_ and silicon oxide was fractured under displacement control, force-displacement response was recorded. The separation energy, adhesion strength and range of the interactions between MoS_2_ and silicon oxide were characterized by analytical and numerical analyses. In addition to the fundamental adhesion properties of MoS_2_, we found that MoS_2_ monolayers on silicon oxide had self-healing properties, meaning that when the separated MoS_2_ and silicon oxide were brought into contact, the interface healed. The self-healing property of MoS_2_ is potentially applicable to the development of new composites or devices using 2D materials.

## Introduction

The extreme flexibility of atomically thin two-dimensional (2D) materials is spurring the development of new types of transparent and flexible electronic devices. In the development of such devices with 2D materials as active components, surface forces are likely to be significant because of the large surface to volume ratio of these materials. In this respect, the adhesion properties of graphene have been considered. The adhesion and separation energies of mono- and few-layer graphene from target or seed copper substrates have been obtained by draping graphene over nanoparticles^1^, blister tests^2–5^, and wedge^6^ and double cantilever beam (DCB) fracture tests^7–9^. While many of these studies have focused on the adhesion or separation energies, potential interaction mechanisms are better identified by making reference to the strength and range of the interactions. The energy, strength and range are embodied in traction-separation relations (TSRs), which are the functional form of the continuum representation of interactions between surfaces. The TSR for the interaction between wet-transferred graphene and silicon was obtained by peeling the graphene from the silicon using wedge tests coupled with numerical analysis^6^. The strength and interaction range of adhesive interactions were, respectively, lower and longer than those normally expected for van der Waals interactions. The interactions between graphene and the diamond probe and graphene and silicon were extracted by displacement-controlled nanoindentation with an ultralow-noise force sensor and an iterative analysis^10^. In this case, the separation energy, strength and range of the interactions between graphene and silicon were more commensurate with van der Waals forces. In contrast to graphene, determining the adhesion behavior of MoS_2_ and other 2D materials is in its infancy.

In this work, we applied fracture mechanics concepts to characterize the adhesion of monolayer MoS_2_. Wafer-scale monolayer MoS_2_ was grown on the silicon oxide surface of silicon strips (8 × 40 mm) by using chemical vapor deposition (CVD)^11^. Once the MoS_2_ had been deposited on the parent silicon strip, fracture specimens were then fabricated by bonding a second silicon strip to the MoS_2_ with an epoxy. The subsequent DCB tests and analysis were used to characterize the adhesion interactions between MoS_2_ and silicon oxide. Interestingly, self-healing behavior was observed between the MoS_2_ and silicon oxide surfaces following delamination.

## Results and Discussion

### Interfacial fracture between monolayer MoS_2_ and SiO_2_

**Figure 1:**
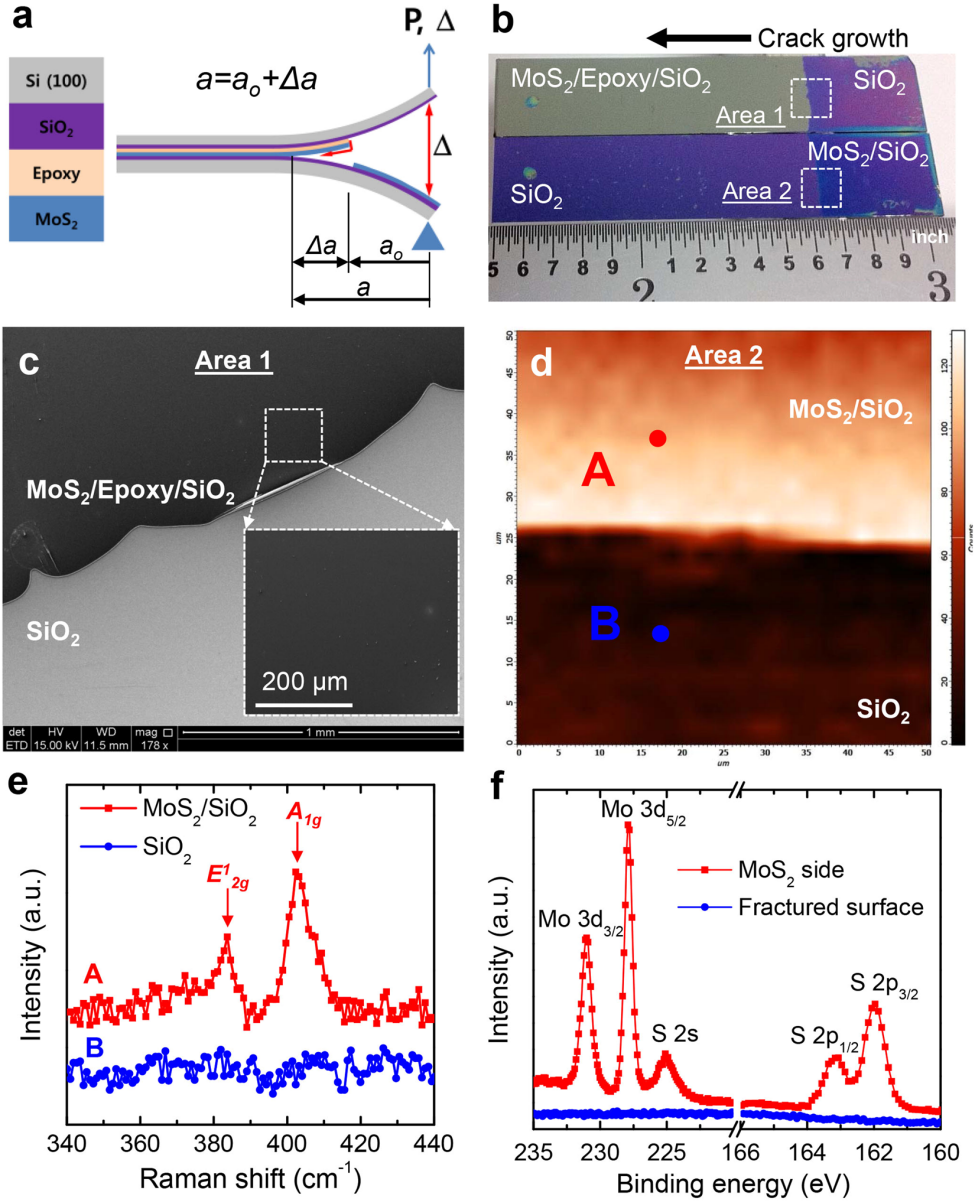
Characterization of the fracture behavior: (**a**) schematic of the fracture experiment, (**b**) plan view of the fracture surfaces of the silicon strips after separation, (**c**) SEM image near the epoxy terminus (Area 1) of the upper strip, (**d**) micro-Raman mapping near the epoxy terminus (Area 2) of the lower strip, (**e**) Raman spectra at points A and B on Area 2 in Fig. 1d and (f) XPS spectra on Area 2 in Fig. 1b.

Large-area monolayer MoS_2_ was synthesized on the silicon oxide surface of  a silicon strip by CVD. Samples having uniform monolayer MoS_2_ were used for the fracture tests after photoluminescence (PL) and Raman observations (see Supporting Information). The layup of the DCB specimen and the loading configuration are identified in Fig. 1a, which also provides a schematic of the delamination path. The fracture surface associated with the bottom silicon strip (Fig. 1b) is characterized by optical microscopy as the purple region associated with bare silicon oxide. The adjacent blue region indicates the presence of MoS_2_ on silicon oxide due to the absence of epoxy there. The complementary regions on the fracture surface of the upper silicon strip were the grey region where the epoxy was covered by MoS_2_ and the purple region corresponding to bare silicon oxide. Furthermore, the fracture surface of the upper silicon strip near the epoxy terminus (Area 1) was imaged (Fig. 1c) by scanning electron microscopy (SEM). The absence of charging in the dark region indicates that the epoxy was completely covered by MoS_2_^8,9^.

Raman spectroscopy was used to check the presence and quality of MoS_2_ in Area 2 identified in Fig. 1b. The Raman map (Fig. 1d) of the integrated intensity of the peak for the ${A}_{1g}$ mode over Area 2 indicates uniformity of transfer. The detailed Raman spectrum (Fig. 1e) taken at point A (Fig. 1d) on the upper fracture surface indicates that the ${E}_{2g}^{1}$ and ${A}_{1g}$ modes of MoS_2_ are positioned at 383.7 and 402.3 cm^−1^, respectively. The difference of the peak positions is approximately 18.6 cm^−1^, indicating the presence of a monolayer^11^. In contrast, both peaks were completely absent at point B on the lower fracture surface, thus meaning that the MoS_2_ was transferred onto the epoxy at that location. The transfer of MoS_2_ onto the epoxy was further characterized with Raman mapping on MoS_2_/epoxy, confirming the complete transfer of MoS_2_ after the fracture tests (see Supporting Information).

X-ray photoelectron spectroscopy (XPS) on Area 2 in Fig. 1b was used to characterize the nature of bonding at the interface between MoS_2_ and silicon oxide (Fig. 1f). On the lower fracture surface, where MoS_2_ was still on the silicon oxide as it had not been in contact with epoxy, there are peaks for Mo 3d_5/2_ and Mo 3d_3/2_ at 227.9 and 231.1 eV, respectively^11^, and additional peaks of S 2p_3/2_ and S 2p_1/2_ associated with doublets of MoS_2_ at 161.9 and 163 eV, respectively^11^. Thus, the XPS spectra suggest that the MoS_2_ on silicon oxide had a 2H-MoS_2_ structure without any metallic components or alloys. The same structure was observed on freshly deposited MoS_2_ on silicon oxide^11^. No such signals were found on the fracture surface, which confirms the observations made by Raman spectroscopy above. In addition, there were no signs of covalent or ionic bonding between silicon and MoS_2_ in the XPS data, which leaves open the possibility of van der Waals bonding. Thus, all four surface characterizations (optical microscopy, SEM, Raman spectroscopy, and XPS) indicate that MoS_2_ was successfully peeled off the silicon oxide surface and transferred to the epoxy.

### Self-healing between MoS_2_ and SiO_2_

**Figure 2:**
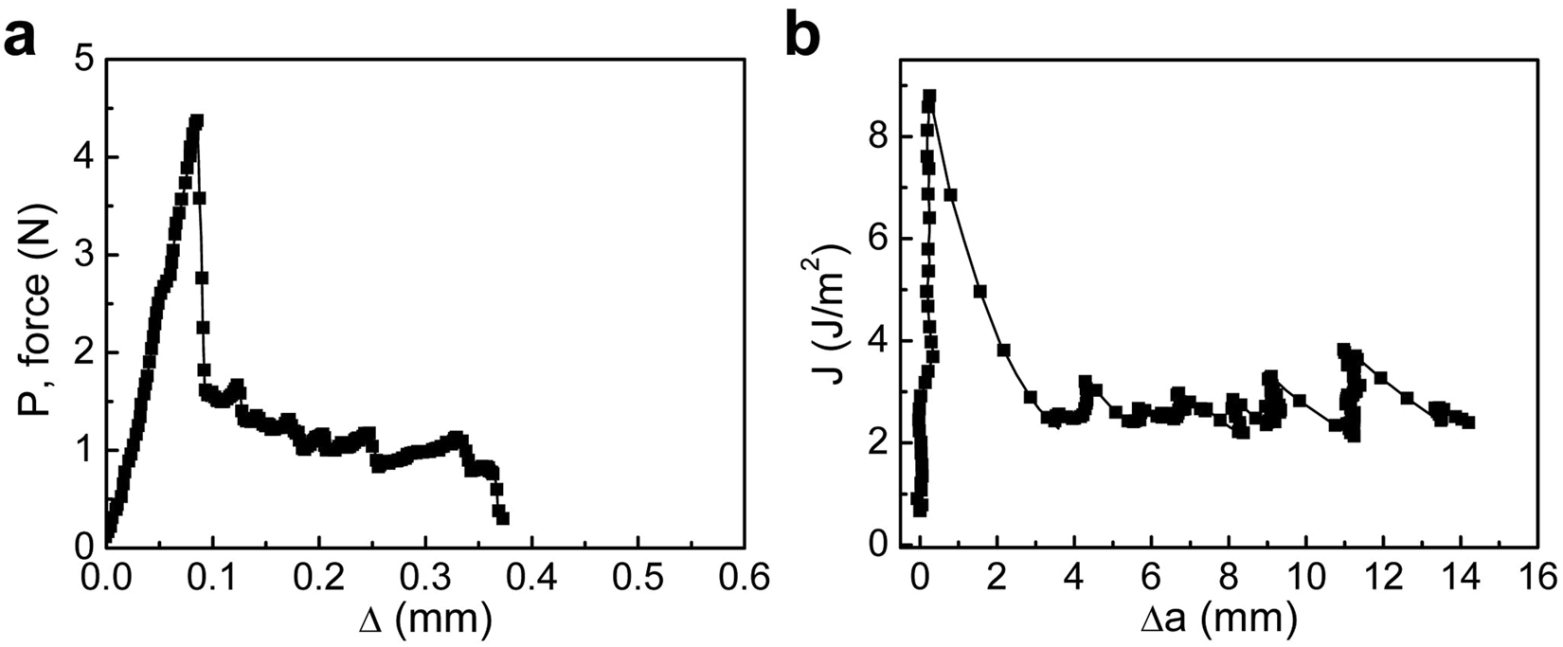
Interfacial fracture behavior of the interface between MoS_2_ and silicon oxide under a ramp load for Sample 1: (**a**) force-displacement response and (**b**) associated fracture resistance curve.

**Figure 3:**
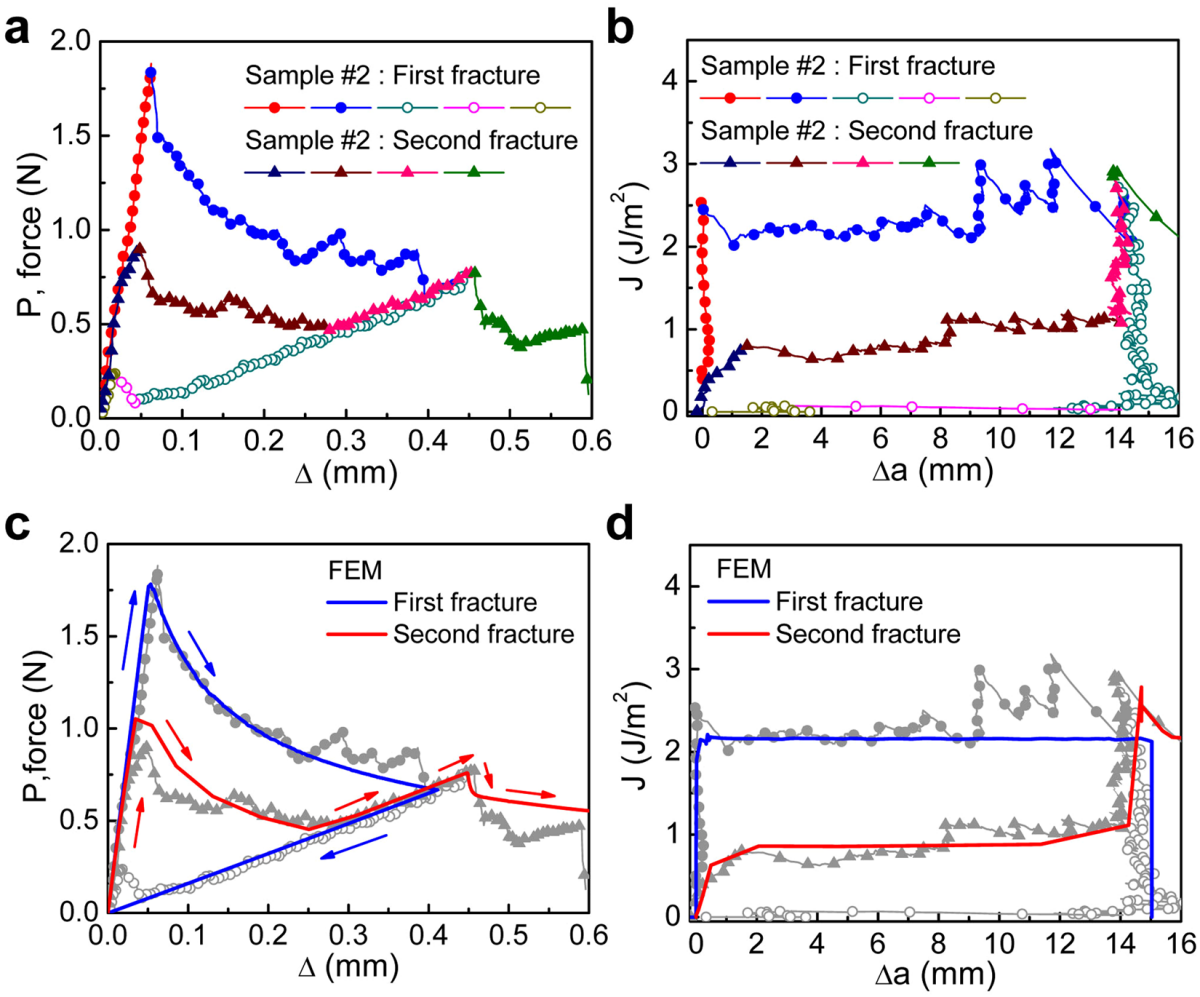
Interfacial fracture behavior of the interface between MoS_2_ and silicon oxide under cycling loads for Sample 2: (**a**) force-displacement responses, (**b**) associated fracture resistance curves, (**c**) comparison of measured and simulated force-displacement responses, and (**d**) comparison of measured and simulated resistance curves.

Figures 2 a and 3a show the force-displacement responses of two samples; Sample 1 was subjected to a ramp in applied displacement (Δ) at 0.042 mm s^−1^ until the MoS_2_ layer completely delaminated from the silicon oxide, while Sample 2 was unloaded and reloaded after a small amount of crack growth on each cycle. The details of the applied displacement history are given in Fig. S5. When Sample 1 was subjected to monotonic loading (Fig. S5b), a linear response ( in Fig. 2a) was observed up to 4.4 N, followed by a sudden force drop associated with crack initiation. Beyond this point, crack propagation was evident in the overall decrease in the load as the applied displacement was increased. The load perturbations in this phase of the response are due to so-called “stick-slip” crack propagation and served to establish the separation energy of the MoS_2_/silicon oxide interface. The test ended with complete separation at an applied displacement of 0.37 mm.

For Sample 2, the linear response ( in Fig. 3a) was more compliant than the one observed in Sample 1 because the initial crack (*a*_0_ in Fig. 1a) was longer. The peak corresponding to the initiation of the delamination between MoS_2_ and silicon oxide was lower at 1.8 N, but the decaying portion of the response (), associated with continued delamination, was similar^9^. This suggests that the fracture energy of the samples was identical. Once the applied displacement reached 0.44 mm, the specimen was unloaded following the profile identified in Fig. S5c. During this time, the force linearly decreased () until the applied displacement was reduced. Upon reloading, the slope of the linear response () remained close to the one observed () under the first load. The force-displacement curves for the first and second loading have the same slope which indicates that the crack length was the same due to healing of the fractured surfaces during the unloading portion of the cycle. This effect has been widely observed in self-healing polymer composites where the force-displacement curve exhibits the same linear increase of force after the fractured composite has healed^12^. However, to best of our knowledge, this observation is the first report on the self-healing in 2D materials either grown on or transferred to silicon oxide.

**Figure 4:**
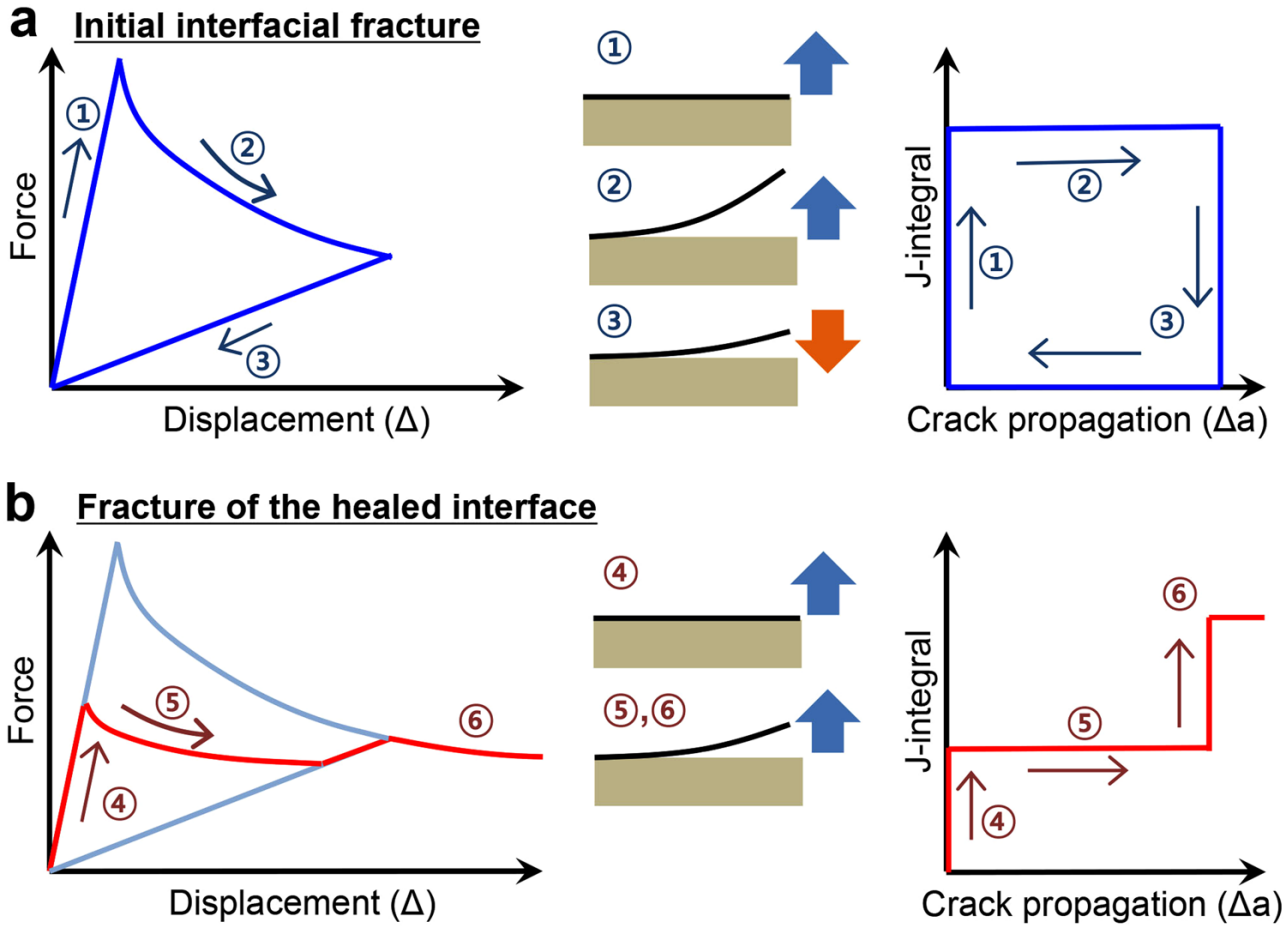
Schematic of the force-displacement responses and fracture resistance curves associated with (**a**) intrinsic and (**b**) healed interfaces. Segments of loading and unloading paths are identified by numbers.

For the second loading, the peak load was only 0.9 N, which is a factor of two lower than the one in the first cycle. Thus, although healing had occurred, it did not completely return the interface to its original strength as will be quantified later. As the applied displacement increased further, the load dropped () as the healed portion of the interface cracked. At an applied displacement of 0.27 mm, the healed portion of the interface was completely separated and the load increased () with a slope corresponding to the crack length at the first unloading. Once the applied displacement returned to 0.47 mm, the load dropped and continued along a path () that suggested that the original interface was again being separated. Figure 4 shows an idealized schematic of this self-healing process in terms of a force-displacement and J-integral responses.

Interestingly, during the unloading portion of the first cycle in Sample 2, as the separation between the crack faces near the crack front became small enough, they were gradually brought into complete contact by adhesive interactions between them as evidenced by the gradual increase () in force to 0.07 N. This was followed and confirmed by the fact that unloading from the 0.07-N load level () to zero followed the same slope as the initial loading (), which is controlled by the initial crack length. Such a response is not common in most fracture experiments but, when it does occur, it means that healing of the fractured surfaces has been activated.

### Analysis of interfacial fracture

In addition to the observation of force-displacement responses, analytical and numerical calculations were used to extract the separation energy, strength and range of the interactions between MoS_2_ and silicon oxide. Because separation and healing were both observed in the experiments, it is important to differentiate between interactions during interfacial fracture (separation) and adhesion (healing). The separation energy associated with each of these interactions was initially extracted using linear fracture mechanics concepts and beam theory. Determining the strength and range of the interactions required numerical methods to handle the functional form of the TSR that are used to represent the interactions.

From simple beam theory, the crack length (*a*) (Fig. 1a) at any point during loading and unloading was obtained from the measured force (*P*) and displacement (Δ) using the following Equation 1.

1 $$P=\frac{Eb{h}^{3}}{8{a}^{3}}{\rm{\Delta }}$$

where *E* was the in-plane Young’s modulus (approximately 129 GPa) of the silicon strips, and *b* and *h* were the width (8 mm) and the thickness (approximately 520 μm) of the silicon strip, respectively. Once the crack length was known for all load levels (see Supporting Information), the J-integral (*J*), which represents the energy available for the creation of a new surface, was determined from the energy release rate (*G*) at the interface with the following Equation 2.

2 $$G=\frac{12{a}^{2}{P}^{2}}{E{h}^{3}{b}^{2}}$$

It also referred to as the resistance to separation and is shown in Figs 2 b and 3b where the J-integral is plotted as a function of the amount of crack growth (Δ*a*). For both samples, the J-integral rose steeply prior to the onset of crack growth. The peak value for Sample 1 was 9 J m^−2^ before dropping sharply to a steady state response that was punctuated by stick-slip behavior. The peak value for the first loading of Sample 2 was 2.3 J m^−2^, which was followed by a much smaller drop to the steady state separation energy. The average value of the steady state separation energy was 2.2 ± 0.1 J m^−2^. The first unloading of Sample 2 resulted in a decrease in the J-integral to 30 mJ m^−2^, while the crack remained stationary. Further reduction of the applied displacement led to an increase in J-integral to an adhesion energy of 70 mJ m^−2^, corresponding to the load jump near the origin in Fig. 3a, which again indicates the initiation of healing. The return to zero applied displacement was then accompanied by a concomitant drop in J-integral.

During reloading, the initial response was similar to the response observed during the first loading, but quickly transitioned to the average separation energy of 0.9 ± 0.16 J m^−2^. Comparing this value to the adhesion energy of 70 mJ m^−2^ that was observed during unloading in the first cycle, it is clear that, while the cracked surfaces were in contact, further healing had occurred. Once the healed crack faces were fully separated, the crack front arrested at the same location where the first unloading had occurred. As the applied displacement was increased, the J-integral rose sharply to approximately 2.8 J m^−2^ when the intrinsic interface again began to separate.

At the continuum level, along with the energies, TSRs can provide more insight on the nature of the adhesion interactions. In this work, a bilinear form (Fig. S6) was assumed for the TSRs and the energy, strength and range of the interactions during separation were extracted in an iterative manner using finite element analysis (see Supporting Information). The best estimates of the strengths and ranges of the interactions are shown in Table 1 for separation of the intrinsic (TSR1) and healed (TSR2) surfaces. The force-displacement responses and delamination resistance curves associated with each of these TSRs are compared (Fig. 3c,d) with the measured responses. Apart from some deviations due to stick-slip behavior, the two sets of force-displacement responses are in very reasonable agreement. The ratio of the separation energies of the healed to the intrinsic interfaces was 0.43. At the same time, the strength of the healed interface dropped by a factor of 10 and the interaction range increased by a factor of 5.

**Table 1:** Values of the parameters in the traction-separation relations for the interfaces between MoS_2_ and silicon oxide.

	TSR 1 (intrinsic)	TSR 2 (healed)
Separation energy (J m^−2^)	2.15	0.9
Stiffness (MPa mm^−1^)	10^6^	10^6^
${\delta }_{n}^{0}$ (nm)	35	3
${\sigma }_{0}$ (MPa)	35	3
${\delta }_{n}^{c}$ (nm)	123	600

The question then arises as to what interaction mechanisms give rise to the TSRs for separation of the intrinsic and healed interfaces as noted in Table 1. Since XPS (Fig. 1f) indicated that there was no covalent bonding between the MoS_2_ and the silicon oxide, the natural expectation is that van der Waals forces would be active. However, compared to van der Waals forces, both the separation energy and interaction range are much larger and the strength is lower. Because of growth of MoS_2_ at the temperature of approximately 700 °C, the intrinsic interaction is not expected to involve capillary effects of water, which can have longer interaction ranges and lower strength^13^. Additional insights as to other interactions will have to be provided by additional spectroscopy such as Fourier Transform Infrared (FTIR) and atomistic simulations. The root-mean-squared (RMS) roughness of the silicon oxide surface before deposition and following transfer was approximately the same at 0.5 nm (Fig. S7). This is much shorter than the interaction range, so it is not clear that the greater separation energy and longer interaction range (compared to van der Waals forces) is due to the roughness of the silicon oxide surface. The separation energy of the healed surface was 43% of that of the intrinsic interaction between MoS_2_ and silicon oxide and the interaction range was approximately five times longer. Since the separation took place in ambient conditions, it is possible that capillary forces associated with water molecules attracted to the fracture surfaces were active for the healing processes. Again, this will have to be clarified by more detailed observations and atomistic simulations^14–17^.

### Repeated healing and separation of the interface

**Figure 5:**
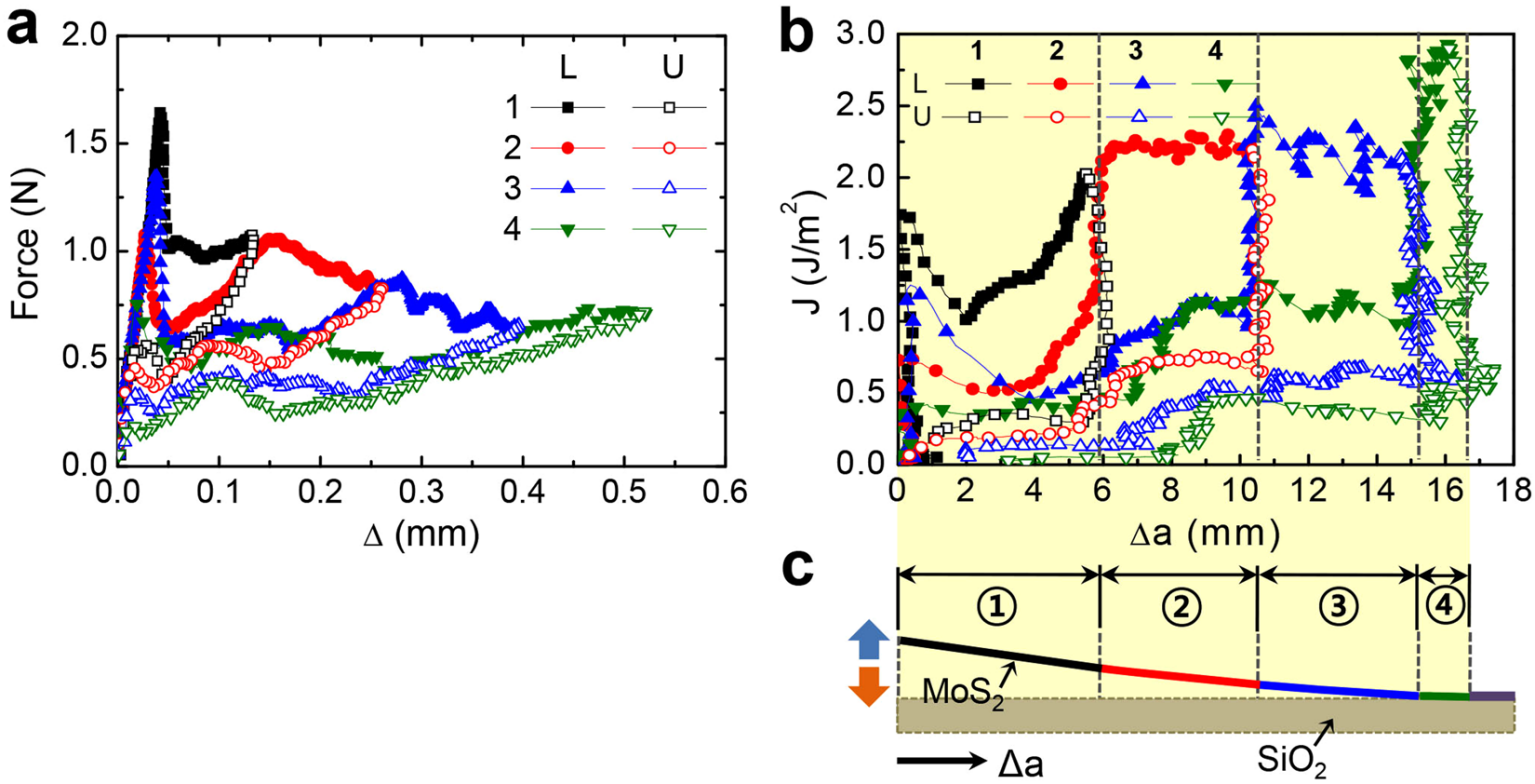
Results from a specimen subject to four continuous loading and unloading cycles: (**a**) force-displacement responses, (**b**) resistance curves and (**c**) identification of the portion of the fractured interface associated with a given load cycle.

**Figure 6:**
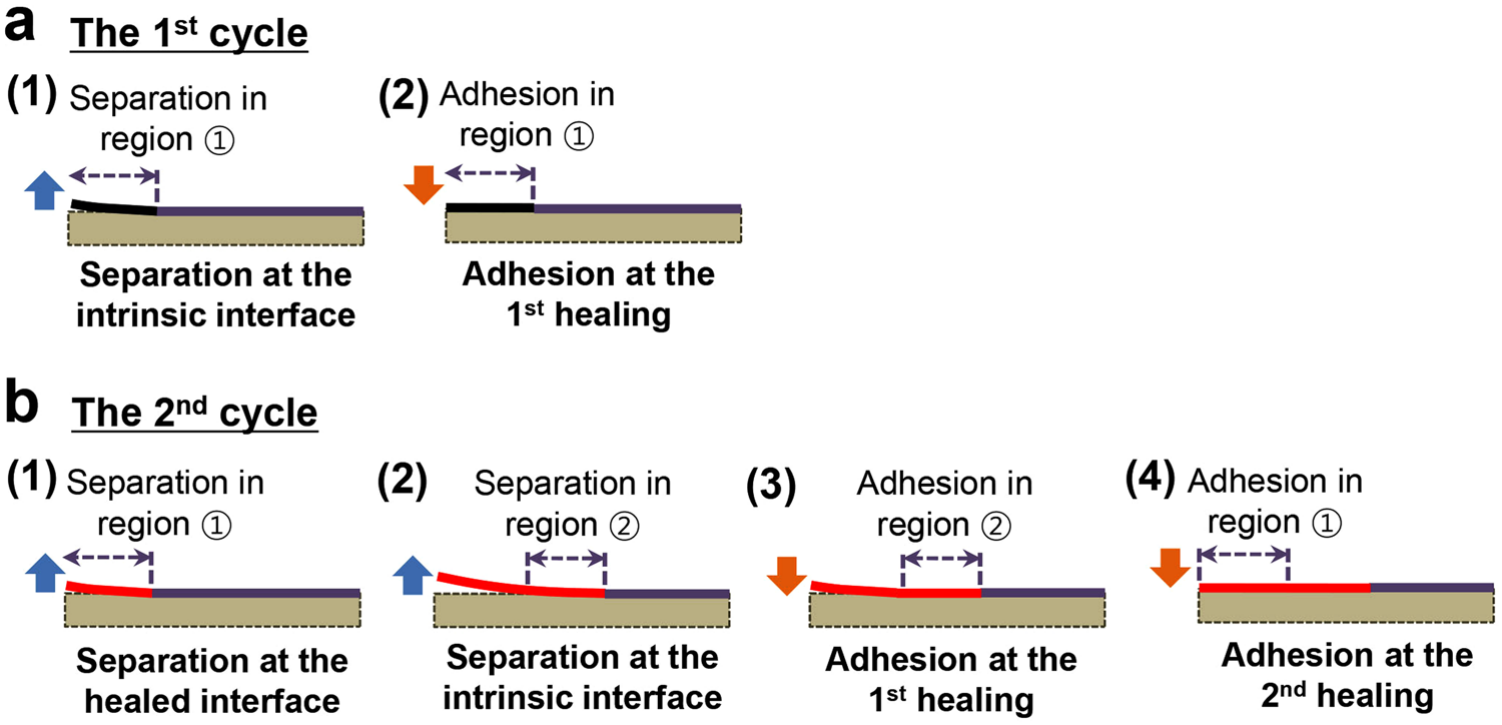
Schematic of the processes related to the separation and adhesion events at different regions of the interface during loading and unloading cycles. The first two cycles in Fig. 4 are described in detail.

The robustness and characteristics of the healing was investigated over four loading and unloading cycles (Fig. 5). The force-displacement responses (Fig. 5a) and associated resistance curves (Fig. 5b) (detailed data for each cycle are shown in Fig. S8) emphasize the contrast between the adhesion energies associated with the intrinsic and healed interfaces. During the cyclic tests, each reloading opened up a new region of the intrinsic interface (labelled ①–④ in Fig. 5c) and each unloading induced adhesion and healing at the fractured interface as described in more detail in Fig. 6. The features are similar to those shown for two loading cycles in Fig. 3. The energies associated with separation and adhesion over the experiment with four cycles are summarized in Table 2. The separation energies for intrinsic interfaces are reasonably consistent from cycle to cycle with an average value of 2.33 ± 0.23 J m^−2^ (Table 2), which is also consistent with the intrinsic separation energy obtained in the experiment shown in Fig. 3. The first unloading cycle encountered healing of region ① (Fig. 5b) with an adhesion energy of 0.29 ± 0.05 J m^−2^. Upon reloading for the second cycle, the healed portion of region ① had an average separation energy of 0.52 ± 0.01 J m^−2^ for a healing efficiency of 26%. A further increase in the applied displacement led to separation of the intrinsic interface (region ②). This pattern of events repeated on each cycle and several features of the healing processes can be observed; (1) the energy required to separate the healed interfaces never reached that of the intrinsic interface. (2) The energy required to separate repeatedly healed surfaces decreased with each cycle. (3) The separation energies increased as the contact time of healed surfaces increased. (4) The adhesion energy associated with each region decreased with each unloading cycle, which might be attributed to increasing amounts of irreparable damage occurring during each separation cycle.

**Table 2:** Summary of the separation energy and the adhesion energy at each cycle: the cells with bold/italic text show the separation energies for the intrinsic interfaces.

	Interaction region	Cycle no.			
		1	2	3	4
Loading: Separation energy (J m^−2^)	➀	***2.03 ± 0.01***	0.52 ± 0.01	0.47 ± 0.01	0.41 ± 0.03
	➁	—	***2.21 ± 0.03***	0.98 ± 0.1	0.89 ± 0.23
	➂	—	—	***2.27 ± 0.03***	1.12 ± 0.09
	➃	—	—	—	***2.78 ± 0.1***
Unloading: Adhesion energy (J m^−2^)	➀	0.29 ± 0.05	0.2 ± 0.01	0.13 ± 0.01	0.05 ± 0.01
	➁	—	0.67 ± 0.09	0.40 ± 0.10	0.24 ± 0.14
	➂	—	—	0.61 ± 0.04	0.38 ± 0.03
	➃	—	—	—	0.61 ± 0.06

## Conclusion

The separation of monolayer MoS_2_ on silicon oxide in DCB fracture tests was purely interfacial, thereby effectively transferring the MoS_2_ to an epoxy. Analysis of the force-displacement responses associated with the intrinsic and healed interfaces established the contrast in the respective adhesion energies as the fracture surfaces were repeatedly separated and brought back into contact. This is a new finding for the adhesive properties of 2D materials and may motivate the development of new composites or devices with self-healing properties.

## Methods

### Preparation and mechanical tests of the DCB samples

High-quality monolayer MoS_2_ was synthesized on silicon oxide on 8 × 40 mm silicon strips by using CVD with MoO_3_ powder and H_2_S gas^11^. A silicon strip of the same size was bonded to the MoS_2_ using a low viscosity epoxy (EP30, Master Bond, Inc.). The laminated beams formed in this way (Fig. S3) were cured at 100 °C for approximately two hours to obtain the maximum adhesion strength of the epoxy layer. Classical DCB fracture experiments (Fig. 1a) were conducted under displacement control while the force and displacement were recorded with a load cell and linear variable differential transformer (LVDT), respectively. Fracture surfaces were observed with optical microscopy, Raman spectroscopy (NT-MDT AFM-Raman spectroscope with a 532 nm laser), XPS (Kratos AXIS Ultra DLD spectrometer), and SEM (FEI Quanta-600).

### Fracture analysis

The force-displacement response of the DCB specimen was recorded by a load cell and LVDT, respectively. Simple beam theory was used to represent the force-displacement of the specimen with the Equation 1. Since *P* and Δ were both measured, Equation 1 allowed the crack length *a* to be estimated. The energy release rate (*G*) at the interface is given by the Equation 2. It allowed the resistance curves to be assembled based on the load and the estimates of crack length. Thus, steady-state response in the resistance curve allowed the estimation of the fracture energy (${{\rm{\Gamma }}}_{ss}$) referred to as the work of separation.

Details of experimental and analysis methods are described in the Supporting Information.

## Electronic supplementary material

Supporting Information
