# Supplementary material for: Adhesion and Self-Healing between Monolayer Molybdenum Disulfide and Silicon Oxide
Source: Sci Rep. 2017 Nov 7;7:14740. doi: 10.1038/s41598-017-14921-9 (PMC5677127; doi:10.1038/s41598-017-14921-9)
Supplement: Supplementary file 1 — Supporting Information [file 41598_2017_14921_MOESM1_ESM.pdf]

Supporting Information

Adhesion and Self-Healing between Monolayer Molybdenum Disulfide and Silicon Oxide

Seung Ryul Na[1†], *Youngchan Kim*[2†], *Changgu Lee*[2,3]*, *Kenneth M. Liechti*[1]* and *Ji Won Suk*[3]*

[1] Department of Aerospace Engineering and Engineering Mechanics, The University of Texas at Austin, Austin, Texas 78712, USA

[2] SKKU Advanced Institute of Nanotechnology, Sungkyunkwan University, Suwon, Gyeonggi-do 16419, Republic of Korea

[3] School of Mechanical Engineering, Sungkyunkwan University, Suwon, Gyeonggi-do 16419, Republic of Korea

[†]These authors contributed equally to this work.

*Corresponding authors: peterlee@skku.edu (C. Lee), kml@mail.utexas.edu (K. M. Liechti) and jwsuk@skku.edu (J. W. Suk)

Keywords: molybdenum disulfide, self-healing, fracture, adhesion, chemical vapor deposition

1. Monolayer MoS_2 on SiO_2

High-quality monolayer MoS_2 was grown on a SiO_2 strip (8 × 40 mm) by using chemical vapor deposition (CVD). The presence and uniformity of the monolayer MoS_2 was confirmed with photoluminescence (PL) and Raman spectroscopy. Figure S1 shows the PL spectrum of the MoS_2 layer on SiO_2. Clear PL peaks are observed at 1.89 and 2.03 eV, which closely match the peak positions of A_1 (trion) and B_1 (exciton) of monolayer MoS_2 described in the literature [1].

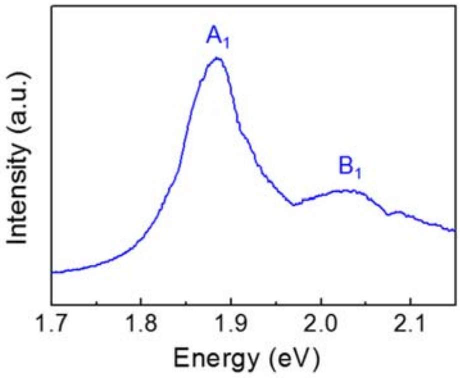

Figure S1. The photoluminescence spectrum of monolayer MoS_2.

In order to prove the homogeneity and uniformity of the CVD-grown MoS_2 sample, we obtained Raman spectra at different positions along the longitudinal direction of the strip (Figure S2a). As shown in Figure S2b, the distances between E^1_{2g} and A_{1g} peaks are 20.4 cm^{-1} for all three positions of the strip, confirming that the film is monolayer MoS_2. Furthermore, Raman maps (50 × 50 μm, 2500 points) of the E^1_{2g} and A_{1g} peak positions were obtained at the center of the strip (using a laser with 532 nm wavelength and 1 mW power). Figures S2c and S2d show that the average peak positions for E^1_{2g} and A_{1g} in the scanned area are 384.2 and 484.6 cm^{-1}, respectively. Based on the PL and Raman observations, it was confirmed that the entire strip was uniformly covered with monolayer MoS_2.

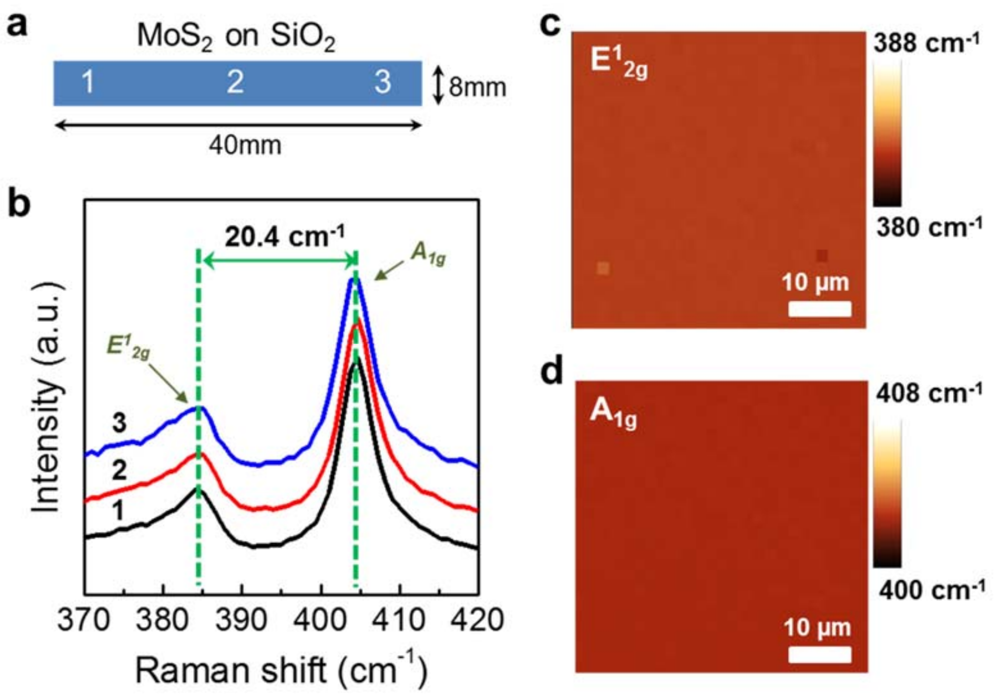

Figure S2. Observation using Raman spectroscopy of monolayer MoS_2 grown on SiO_2. (a) Schematic of the growth substrate and measurement positions for Raman observations. (b) Raman spectra of MoS_2 at different positions in the sample strip. (c and d) Raman maps of E^1_{2g} and A_{1g} peak positions.

2. Preparation of the double cantilever beam fracture samples

Once high-quality and monolayer MoS_2 was successfully synthesized on a silicon oxide strip, the other silicon oxide strip was partially coated with uncured epoxy (EP30, Master Bond, Inc.) in order to fabricate the double cantilever beam (DCB) specimen. The epoxy coated silicon oxide strip was placed on the MoS_2 surface (Figure S3a). The DCB specimen (cross section is Si/SiO_2/MoS_2/epoxy/SiO_2/Si) was cured at 100 ºC for two hours (Figure S3b). The epoxy terminus creates the initial crack of length (a_0) which ranged from 1 cm to 2 cm. Finally, aluminum loading fixtures were bonded to DCB specimens (Figure S3c)

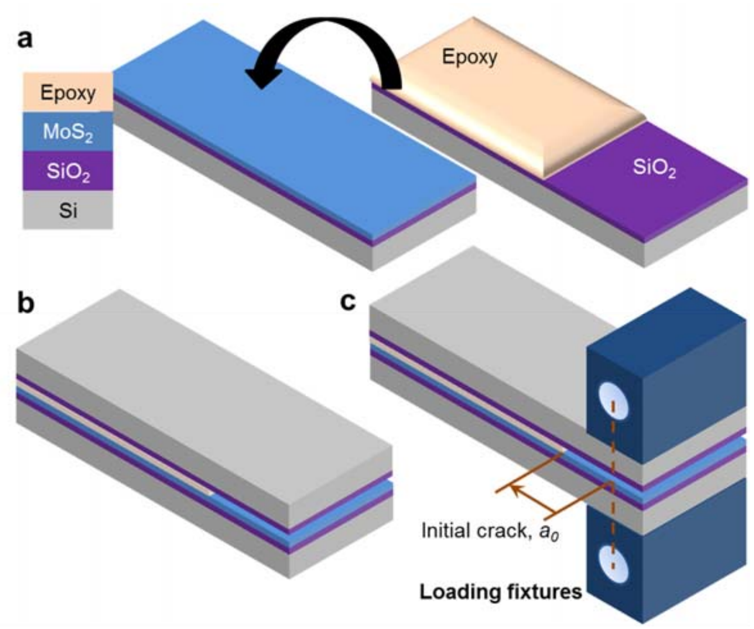

Figure S3. Schematic of double cantilever beam specimens: (a) bonding process, (b) curing at 100 ºC for two hours, and (c) attaching the loading fixtures.

3. Monolayer MoS_2 transferred on epoxy

As long as the silicon strip with MoS_2 was properly bonded to the second silicon strip with a thin epoxy layer, the MoS_2 was completely transferred to the epoxy layer. In order to confirm the complete transfer of MoS_2 onto the epoxy, the transferred MoS_2 on the epoxy side was checked with Raman spectroscopy after the fracture test (Figure S4a). Figure S4b shows the Raman spectra obtained from the growth substrate S1 and the transferred substrate S2. Two peaks for the E^1_{2g} and A_{1g} are present at 383 and 404 cm^{-1} for both substrates, respectively. The presence of the E^1_{2g} and A_{1g} peaks for the substrate S2 explicitly proves that monolayer of MoS_2 on the silicon oxide was successfully transferred to the epoxy surface. Furthermore, there is no strain effect on the MoS_2 layer due to the fact that the characteristic peaks were identical before and after transfer [2].

To check the uniformity of the transferred MoS_2, several areas of the substrates S1 and S2 were characterized by Raman mapping of the peak intensity of the A_{1g}. After transfer, Raman peak intensity

(A_{1g}) of the MoS_2 at the region (3) of the substrate S1 has almost a trivial value as shown in Figure S4c, which indicates that the MoS_2 was completely detached from SiO_2. On the contrary, the epoxy regions (5) and (6) of the substrate S2 present clear presence of Raman peak intensity (A_{1g}) of the MoS_2 film as shown in Figure S4d. These results prove that the MoS_2 was perfectly transferred onto the epoxy during the fracture experiment.

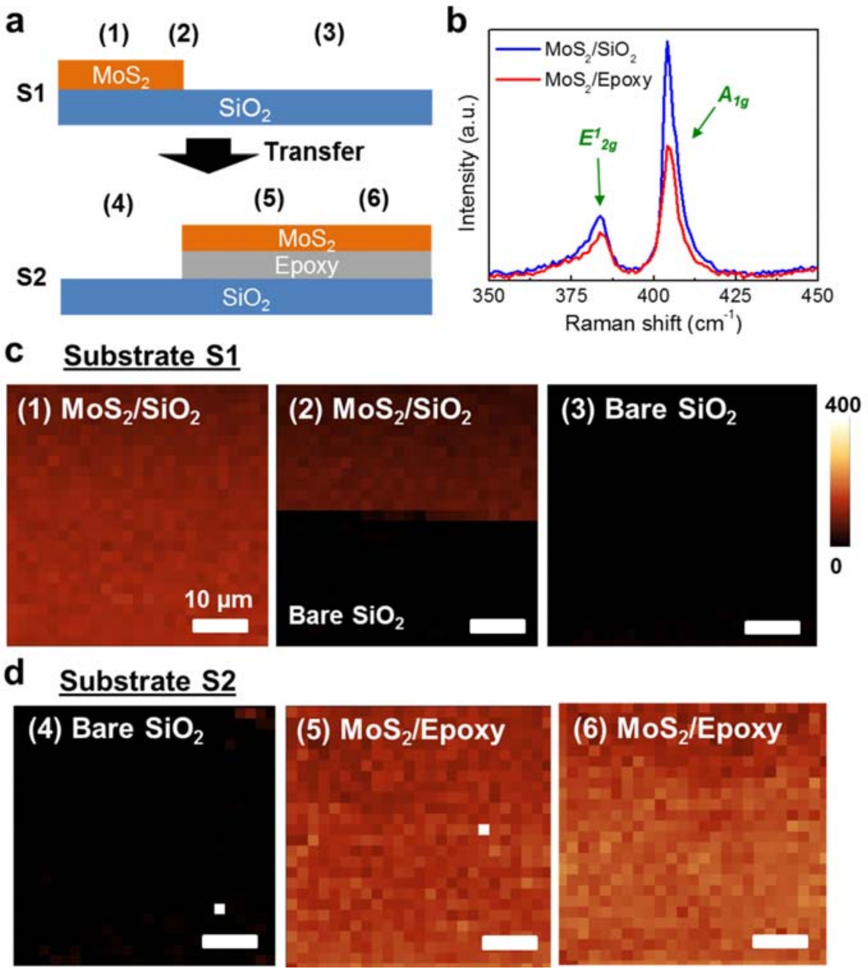

Figure S4. (a) Schematic of the transfer of monolayer MoS_2 by delamination. (b) Raman spectra of MoS_2 before and after the fracture test. (c and d) Raman maps of A_{1g} peak intensity of MoS_2 at different positions of the substrates S1 and S2 described in Figure S4a.

4. Applied displacement profiles

In all the experiments that were conducted, the separation of the silicon strips was controlled by specifying the displacements applied at the point of application of the load. The time history of a general case is shown schematically in Figure S5a. The initial loading is shown in blue, where the applied displacement rate was constant and sufficient to produce separation of the intrinsic interface. Once steady state growth of the crack had occurred, the applied displacement was returned to zero at the same rate as the loading portion. In some cases the loading was reapplied (red) at the same rate but to a higher level, resulting in the separation of any healed region from the previous unloading as well as crack growth along a new portion of the intrinsic interface, whereupon the specimen was again unloaded. As many as four cycles were applied, with increasingly larger maximum applied displacements. The circled numbers correspond to different regions of the interface that were healed or separated, making reference to discussion related to Figure 4.

Figure S5b corresponds to a case where the initial ramp in displacement was applied until the specimen had completely separated. Figure S5c reflects unloading and reloading of a specimen.

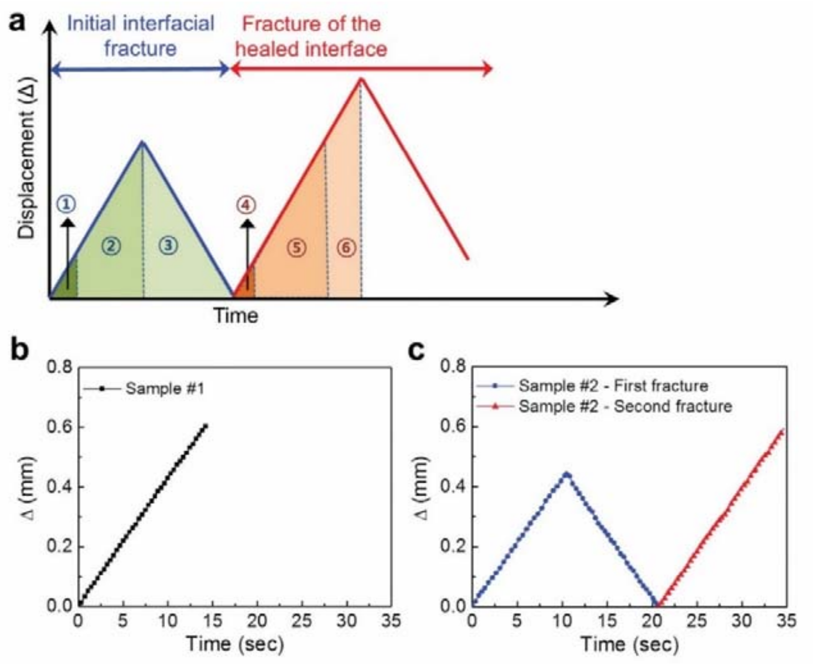

Figure S5. Displacement profiles of the monotonic and cyclic loading at a rate of 0.042 mm s^{-1}: (a) the schematic of a general load history, (b) a single ramp load and (c) a cyclic load.

5. Traction-separation relations

Interactions during separation are characterized by their fracture energy, strength, and interaction range. Although simple beam theory is sufficient for characterizing the fracture energy, further details of the interaction must be determined by finite element analysis (FEA). The continuum description of the adhesion interaction between two surfaces is often expressed as the traction-separation relation. Although many functional forms are possible, one of the simplest, the bilinear form (Figure S6) was selected for this study.

Bilinear traction-separation relations are defined by five key-parameters including stiffness (K), strength (σ_0), damage initiation (δ_n^0), critical separation distance (δ_n^c) and the steady-state fracture energy (Γ_{ss}). The first four parameters were determined by iteration until simulated force-displacement responses were in good agreement with the measured ones. First, the silicon oxide strips (8 × 40 mm), and

the layer of epoxy were modeled by continuum plane strain four node elements with reduced integration (CPE4R element in ABAQUS). The constitutive model for silicon oxide and epoxy were assumed to be linearly elastic, homogeneous, and isotropic material with the moduli of 129 GPa and 3 GPa, respectively. Also, the Poisson's ratios in silicon oxide and epoxy were 0.23 and 0.45, respectively.

The thickness of the MoS_2 layer is approximately 0.7 nm, making it unnecessary to model it explicitly. Nonetheless, the interface between MoS_2 and the silicon oxide was modeled by the bilinear traction-separation relation

$$\sigma = K\delta_n H\left(\delta_n^0 - \delta_n\right) + \left[1 - \frac{\delta_n^c\left(\delta_n - \delta_n^0\right)}{\delta_n\left(\delta_n^c - \delta_n^0\right)}\right] K\delta_n H\left(\delta_n - \delta_n^0\right) \tag{S1}$$

where $H(\delta_n)$ is the Heaviside function. It should be noted that the steady-state fracture energy ($\Gamma_{ss} = \frac{1}{2}K\delta_n^0\delta_n^c$) is the area underneath the traction-separation relation shown in Figure S6. Thus, this model allowed initiation and growth of interfacial fracture to simulate the peeling of the MoS_2 layer from the silicon strip. Finally, it should be noted that the simulation of unloading was unable to account for the healing that was observed in the experiments. This was due to unexpected complications that remain unresolved at this time.

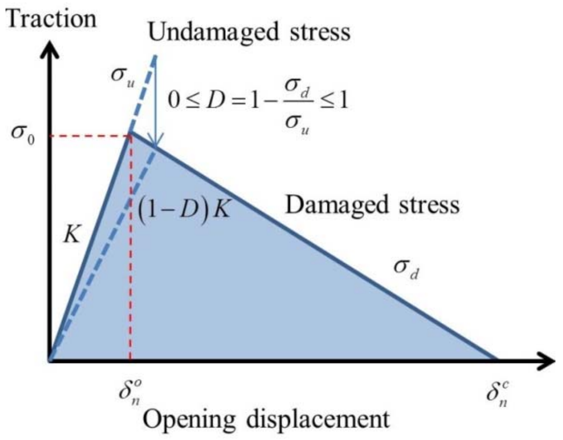

Figure S6. Schematic of a bilinear traction-separation relation.

6. Roughness of MoS_2

The atomic force microscope images (Figures S7a and S7b) represent the morphology of MoS_2 on silicon oxide after deposition and on epoxy after delamination experiments, respectively. The root-mean-squared (RMS) roughness on both surfaces was approximately 0.49 nm and 0.59 nm, respectively. The roughness of MoS_2 on the silicon oxide is close to the roughness of the mirror-polished silicon surfaces on which it was grown. The similarity in roughness before and after transfer indicates that the epoxy was able to conform closely to the MoS_2 surface.

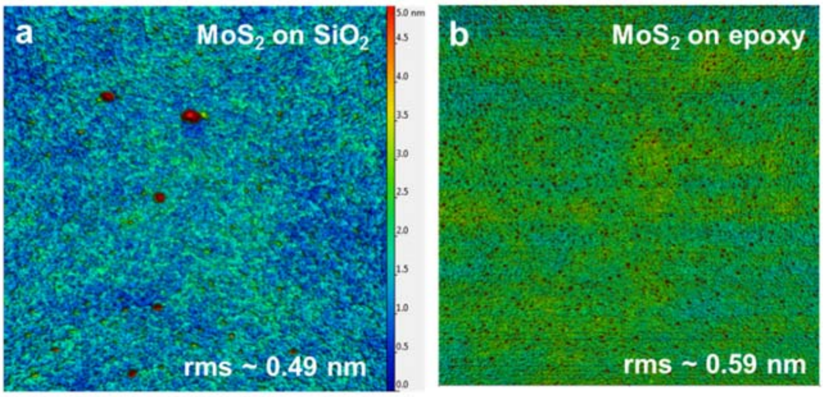

Figure S7. The roughness of (a) as-grown MoS_2 on SiO_2 and (b) transferred MoS_2 on epoxy.

7. Multi-cycle loads

The initial observation of healing (Figure 3) motivated the examination of the effect of repeated healing and separation using multi-cycle loads (Figures 5 and S8). The force-displacement response in the left column represents the response of *i*-th load cycle (i =1, 2, 3, and 4), with its corresponding resistance curve in the right column. No matter which cycle load was selected, its fracture resistance curve exhibited the characteristic response shown in Figure 4.

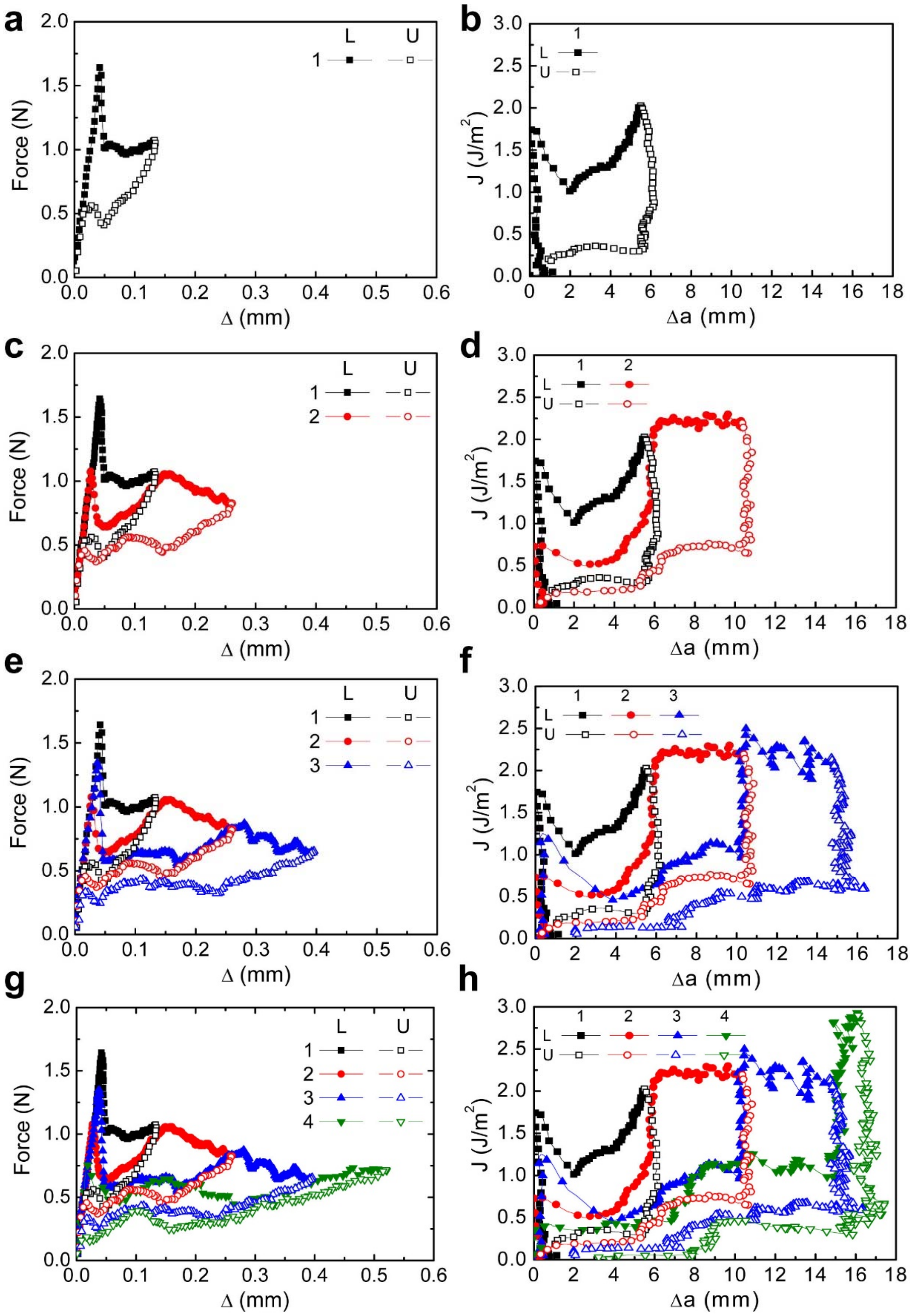

Figure S8. The force-displacement and its fracture resistance plots during cyclic tests.

References

[1] K. F. Mak, K. He, C. Lee, G. H. Lee, J. Hone, T. F. Heinz and J. Shan, *Nature Materials*, 2013, **12**, 207-211.

[2] Y. Kim, H. Bark, G. H. Ryu, Z. Lee and C. Lee, *Journal of Physics: Condensed Matter*, 2016, **28**, 184002.
